# Practicing outcome-based medical care using pragmatic care trials

**DOI:** 10.1186/s13063-020-04829-7

**Published:** 2020-10-29

**Authors:** Tim E. Darsaut, Jean Raymond

**Affiliations:** 1 Department of Surgery, Division of Neurosurgery, University of Alberta Hospital, Mackenzie Health Sciences Centre, 8440 - 112 Street, Edmonton, Alberta T6G 2B7 Canada; 2 Department of Radiology, Service of Interventional Neuroradiology, Centre Hospitalier de l’Université de Montréal – CHUM, 1000 Saint-Denis street, room D03-5462B, Montreal, QC H2X 0C1 Canada

**Keywords:** Randomized trials, Pragmatic trials, Clinical research methodology, Medical ethics, Research ethics

## Abstract

The current separation between medical research and care is an obstacle to essential aspects of good medical practice: the verification that care interventions actually deliver the good outcomes they promise, and the use of scientific methods to optimize care under uncertainty. Pragmatic care trials have been designed to address these problems. Care trials are all-inclusive randomized trials integrated into care. Every item of trial design is selected in the best medical interest of participating patients. Care trials can eventually show what constitutes good medical practice based on patient outcomes. In the meantime, care trials give clinicians and patients the scientific methods necessary for optimization of medical care when no one really knows what to do.

We report the progress of 9 randomized care trials that were used to guide the endovascular or surgical management of 1212 patients with acute stroke, intracranial aneurysms, and arteriovenous malformations in a single center in an elective or acute care context. Care trials were used to address long-standing dilemmas regarding rival medical, surgical, or endovascular management options or to offer innovative instead of standard treatments. The trial methodology, by replacing unrepeatable treatment decisions by 1:1 randomized allocation whenever reliable knowledge was not available, had an immediate impact, transforming unverifiable dogmatic medical practice into verifiable outcome-based medical care. We believe the approach is applicable to all medical or surgical domains, but widespread adoption may require the revision of many currently prevalent views regarding the role of research in clinical practice.

## The problem of the current research-care separation

Whether innovative or commonly used medical tests or interventions actually deliver the good outcomes they promise should be a central concern of clinical medicine. Yet, such questions and their answers have been relegated to clinical research since the Belmont report, in which research and practice are regarded to be distinct activities [1]. According to the Belmont demarcation, clinical research and trials are experiments that use patients to gain “generalizable knowledge.” This goal then differs from medical practice, an activity that can use any intervention, including an innovation “that has a reasonable chance of success,” provided the intent is to benefit the patient. This view creates a gulf or disconnection between the provision of care and the evaluation of the results of care. In addition, it places science and ethics in opposition; by reserving trial methods to “research” conceived outside care, the demarcation leaves medical practice bereft of scientific guidance. In practice, the research-care separation encourages case-by-case human experimentation without methods within care. This is how we have come to practice unverifiable medicine on a large scale, as exemplified by wide variations in the use of medical interventions, overdiagnosis, and overtreatment [2–4].

How are we to provide reliable medical care when no one knows what to do? When and how are we to verify the results of care interventions? Clinical trials can provide a solution to these two problems, but they must be designed accordingly.

Our goal is to share our ongoing experience using the care trial methodology to bridge the gap created by the research-care demarcation and to guide medical practice under pervasive uncertainty. We first present how explanatory and pragmatic trials differ. We then discuss the pragmatic care trial methodology, explain the clinical context that inspired this solution, and share our experience with nine ongoing care trials in neurovascular care. Problems we have encountered related to participation, funding, and publication are finally reviewed.

## Trial designs

Outcome-based medical care can be defined when the results of care are rigorously assessed using scientific methods provided by clinical trials. However, not all trial designs will do; pragmatic trials are more appropriate for this task than explanatory trials. There is still much confusion and contemporary authors often prefer to describe a spectrum of trial designs [5, 6], but the difference in methodologies between pragmatic and explanatory trials was delineated more than 50 years ago by Schwartz and Lellouch [7] who were very clear about the ethical meaning of the distinction: “Fundamental research aimed at the verification of a biological hypothesis is done on a…population which is ultimately treated as means rather than as an end…Normally, explanatory work must be done on animals, therapeutic trials on human subjects being limited to pragmatic experiments” [7].

From the logic of experimentation, explanatory trials resemble laboratory work. Explanatory trials are artificial experiments performed in research subjects selected to reveal a “signal” or to prove a mechanism that may not otherwise be visible in normal conditions. Negative explanatory trials are useful to refute a mechanistic hypothesis: if an experimental treatment does not work under such favorable conditions, then it is safe to say it will not work in practice. But the results of a positive explanatory trial obtained in artificial settings in selected patients cannot be generalized to justify the clinical use of a new treatment. On the contrary, pragmatic trials assess the results of treatments practiced in the reality of medical care, given the diversity of patients, operators, and clinical contexts. A positive pragmatic trial can be generalized to justify the clinical use of a treatment [8].

Unfortunately, explanatory designs are still too often preferred by research agencies and Industry because they are thought to be more efficient or cost-effective to gain knowledge or to show treatment in a good light [9–12]. Pragmatic trials continue to be obstructed by multiple obstacles and are thus difficult to conduct [13, 14].

## Pragmatic care trials

Struggling with a pragmatic trial that did not progress efficiently [13, 14], we came to realize that many misconceptions regarding research and practice would need to be revised if we wanted to practice outcome-based medical care [15]. Pursuing Schwartz and Lellouch’s reflection, we went one step further and proposed, with the help of experienced thinkers in trials and ethics (Doug Altman and David Roy), that certain pragmatic trials, or “care trials,” could be conceived and designed as optimal care in the presence of uncertainty [15, 16]. Care trials are thus a specific sort of pragmatic trial that reintroduce scientific norms in medical practice, but work under the overarching rule of care ethics: every item of the trial design must be selected in the best medical interest of the patient [16].

Briefly, outcome-based medical care and medical care ethics both require a way to define what constitutes good medical practice; they need “validated care.” They also need a fundamental distinction between validated and unvalidated care, for the two types of care must be practiced differently. Medical care and ethics require not only a way to define what “good medical practice” will turn out to be (at the end of the enquiry), but also what good practice should be in the meantime (while no one yet knows what to do). Care trials can play a dual role in guiding outcome-based medical care in the presence of serious uncertainty: they are the test that a medical intervention must pass in order to qualify as “validated care,” defined as care proven to improve patient outcomes. In the meantime, they are the ethical way to practice yet-to-be validated care when knowledge about best practice is lacking. Care trials are pragmatic randomized trials integrated into a real-world medical or surgical practice that they then regulate. The central principle of all care trials is that when a care intervention (surgical, endovascular, or medical—anything that carries risk) is proposed, and that the intervention has never been proven to improve patient outcomes, it cannot be offered or practiced in the same manner as normal, validated care. The clinical uncertainty cannot wait for reliable knowledge: it requires an immediate change in the way care is provided. Care must be provided under a pragmatic research protocol integrated into practice, where each item of the protocol is chosen to optimize benefits and minimize harm in the best medical interest of participating patients. Gaining knowledge that might be of benefit to other future patients is an important but secondary objective of a care trial, one that cannot be assured upfront (as research funders and institutions may wish). Pragmatic care trials are all-inclusive, treatment protocols are flexible and adaptable to particular patients, tests and follow-up visits are standard and routinely used, with no extra cost or risk.

## The context

Before we explain the progress that has been achieved in implementing this idea, we must say a few words about the clinical context of our practice. Endovascular treatments have revolutionized the management of common neurovascular diseases such as intracranial aneurysms and ischemic stroke. These advances qualify as progress because they have convincingly been shown to improve patient outcomes in pragmatic trials [17, 18]. Many other technological advances have changed practices, along with escalating costs, but no one knows if for better or for worse. Many endovascular treatments are widely practiced without evidence they are beneficial [19]. Endovascular treatments have often replaced surgical treatments on the unverified assumption that they were less risky; others have been used on a large scale without evidence they were even safe and effective. This has not always been the case, as some trials that have finally been conducted have shown [20–22]. Neurovascular interventions remain for the most part unsupported by reliable evidence. Clinical neurovascular research is still mostly limited to retrospective case series and observational studies [23–30]. Even regulatory authorities rely on case series of at most 100 carefully selected patients to approve new devices [23, 31]. Trials in our field are infrequently carried out, and when they are, their design is almost always too explanatory to properly inform clinical practice [32, 33].

## The solution

Care trials can be a remedy to ongoing unverifiable medical care and to irrelevant or misguided explanatory clinical research [14]. They are the way to prudently offer promising but unvalidated innovative devices and interventions instead of standard treatments to patients for whom standard treatments seem less promising, or to objectively allocate rival management options when no one really knows which one is best, in clinical contexts where outcome-based medical care can now be defined and verified.

We have designed and launched nine care trials to help clinicians provide neurovascular care under uncertainty in real-time [34–40]. These nine trials are now in routine use in 3 Canadian centers, with one trial ongoing in multiple centers in France. A total of 1212 neurovascular patients have thus far been recruited in various care trials in a single center (Montreal). Other participating centers have contributed 924 patients. Progress reports for 5 of the care trials have been published [41–44] to show they can be done and to encourage additional participation [43]. The various care trials, registration numbers, funding, number of patients enrolled in each trial, the status of each trial, and corresponding publications are summarized in Table 1.

**Table 1:** Active care trials integrated into routine care

Care trial	Registration number	Trial concerns	Number of enrolled patients^a^	Corresponding publications
CURES	NCT01139892	Surgery or endovascular for unruptured aneurysms	189	[34, 41]
ISAT-2	NCT01668563	Surgery or endovascular for ruptured aneurysms	123	[40, 45]
FIAT	NCT01349582	Flow diversion or best standard care for aneurysms	217	[35, 42]
STAT	NCT01340612	Stent or best standard care for aneurysms	126	[36]
TOBAS	NCT02098252	Arteriovenous malformations (AVMs), treat or not	205	[37, 44]
		Pre-op embolization or not		
TATAM	NCT03691870	AVMs, transvenous embolization or best standard care	8	[38]
EASI	NCT02157532	Acute stroke, mechanical thrombectomy or best standard care	320	[33, 46, 47]
RISE	NCT03936647	Intra-saccular flow diverters or best standard care for aneurysms	9	[39]
CAM	NCT04155606	Management of unruptured aneurysms	15	[48]
**Total**			1212	

^a^At CHUM as of March 10, 2020

## How care trials are used in practice

With the exception of trials that address management uncertainties in urgent circumstances, care trial inclusions are made during a weekly multidisciplinary (neurology, neurosurgery, neuroradiology) meeting which reviews all patients with unruptured intracranial aneurysms and ruptured or unruptured brain arteriovenous malformations (AVMs), for whom diverging opinions had previously been the rule rather than the exception. The use of the care trial methodology in the management of these patients is now systematic, for the trials are designed to be all-inclusive and pre-randomization is accepted in five care trial protocols [49]. Pre-randomization has resulted in few cross-overs thus far (in the range of 5–10%). The clinical use of unvalidated endovascular innovations such as flow diversion or stenting in the treatment of aneurysms, or the venous approach to AVM embolization, are now systematically restricted to a care trial protocol [35, 36, 38, 39].

The web-based platform which ensures that randomized allocation can be performed without delay on any hospital computer at any time has facilitated the use of the care trial methodology in the management of patients with urgent conditions. The International Subarachnoid Aneurysm Trial-2 (ISAT-2), comparing surgical clipping and endovascular treatment, is commonly but variably used by individual clinicians involved in the treatment of ruptured aneurysms [40], while the Endovascular Acute Stroke Interventions (EASI) trial, which assesses thrombectomy and which was initially systematically used prior to the publication of positive trials, is ongoing for those acute stroke patients who do not match the selection criteria of previous RCTs, as well as for patients with tandem (cervical and intracranial) arterial occlusions [46].

Electronic case report forms (CRF) are simple and concise, and these are most often completed by participating clinicians and neurovascular fellows at the end of surgical or endovascular interventions, at the time of discharge or routine clinical follow-up visits. Each CRF takes less than a minute to complete.

## Difficulties

While our primary objective is to communicate the progress we have achieved and how it has transformed our daily medical practice, we also want to report lessons we have learned as we worked out how to apply the care trial approach in reality. We will now review the difficulties that remain to be addressed.

Integrating a pragmatic trial into medical practice requires adjustments on both the research and care fronts: on the one hand, the trial design must be adapted to offer care in the best interest of each patient, while clinical practice must also be disciplined to transparently acknowledge the current uncertainty and to modify the medical or surgical action accordingly. Unsurprisingly, care trials have been met with resistance from both sides: the pragmatic design adapted to care has been criticized by conventional trialists and the randomized allocation that protects patients from the preventable harm related to prescribing unvalidated interventions remains poorly accepted by the community of clinicians and patients [47, 50–53]. Many physicians declined participation or did not enroll patients because they preferred to use case-by-case reasoning rather than submit patients to randomized allocation of treatment options, and many patients refuse participation because they wanted their doctor to “rise above the uncertainty” and choose the best option for their particular circumstances. The identification of eligible patients in multidisciplinary consultations and the use of an algorithmic process of treatment allocation which combines clinical judgment and pre-randomization has somewhat tempered these concerns, with improved recruitment in some trials [44]. Other strategies (such as “trials within cohort”) may be more appropriate in other clinical circumstances [54].

The care trial methodology and the specific studies in question have been presented in innumerable national and international meetings, but care trials have caught on in only a small number of centers. Participation is commonly declined by initially interested centers after they recognize the lack of funding. Hospital administrators have repeatedly voiced concerns that care resources and hospital budgets were being diverted for research purposes, or that special insurance coverage was necessary for care research. Concerns from research ethics committees typically revolved around the recruitment of patients by attending physicians, concerns about “equipoise” variously interpreted, and around trial design aspects that were judged too pragmatic. The unclear separation between research and care was a frequent criticism.

## Funding

We have had little success in promoting the care trial methodology in many centers organized in such a fashion that implementing a trial protocol without financial support is inconceivable. This is why we have repeatedly attempted to obtain financial support, but without success. Except for one trial comparing surgical clipping and endovascular treatment of unruptured aneurysms, the Collaborative Unruptured Aneurysm Endovascular vs Surgery (CURES) trial, which was initially (but no longer) supported by the Canadian Institutes of Health Research, no care trial has been financed by public institutions or Industry. The costs related to the design and use of the web-based platforms have been covered by departmental seed grants for education and research. We have repeatedly applied for financial support of multiple care trials to Canadian and French research agencies or to Industry, but grant applications were rejected. The most frequently evoked reasons for rejection were doubts regarding feasibility and criticisms regarding the pragmatic nature of the trial design, such as insufficient selection of patients or of participating physicians or centers, treatment protocols being too flexible, and lack of blinding of the care personnel involved in the trial.

On one hand, research agencies are primarily interested in supporting research projects that “can advance knowledge” to enhance public health or control diseases and their consequences. They are committed “to ensure continued high return on the public investment in research” (www.nih.gov/about-nih/what-we-do/mission-goals). On the other hand, if the care trial methodology is a way to practice medicine in the presence of uncertainty, then is it really “research”? Many research funding agencies see no reason why they should pay for the optimization of care. At the same time, the primary objective of research agencies, to “gain scientific knowledge,” may explain their common preference for the explanatory methodology [55]. In a fundamental way, research agencies pay for data, and RCTs, particularly for common surgical diseases, are reputedly difficult to conduct. Thus funding agencies commonly place considerable weight on the concept of “feasibility,” but refusing funding because of infeasibility is a self-fulfilling prophecy. The real question, we believe, is not whether these necessary trials are feasible and whether they should be attempted at all, but how *can* they be done, for they must be done.

In a similar vein, when the care trial pertains to a novel treatment or device, then public funding agencies are reluctant to pay for what they see as the responsibility of Industry, which is expected to finance the research showing the benefits of the products it wants to sell. In turn, the Industry, which is well-capable of designing RCTs for drugs, does not bother to engage in rigorous clinical research when not formally required by regulatory authorities. Without the need to rigorously verify the clinical value of new devices, the medical community is deprived of an important incentive and of the financial resources to properly test new treatments and devices.

While we could argue that verifying whether our treatments do more good than harm should be a duty for all clinicians, especially when we want to offer a novel intervention, or that these expensive treatments are sufficiently reimbursed and our services sufficiently well paid to cover the cost of that important verification, we understand that until the methodology is widely accepted and seamlessly integrated to care, participation of many centers will depend on monetary compensation.

## Publications

Manuscripts reporting our accumulating experience with care trials have repeatedly been turned down before eventual acceptance for publication [35, 41, 43–45], in spite of being rigorously reported using the CONSORT statement [45, 56]. A detailed example of criticisms from reviewers has been published [47]. Many reviewers were concerned by the participating centers’ and physicians’ expertise, claiming that poor selection of clinicians could explain trial results that were not as good as results obtained in previously published (but very likely biased) case series. Others were concerned about the publication of interim results [55], fearing that the community would incorrectly conclude that our preliminary work would be interpreted to mean that there was no difference between the treatments under study [57].

## Personal care, equipoise, and feasibility

The notions of personalized care and equipoise loom large in the resistance to care trials we have encountered. One major difficulty since Fried [58] is the mistaken idea that by participating in the trial doctors must relinquish their clinical judgment to choose the best treatment and that the patient is thus denied individualized, personalized care. This is a mistake, because clinical judgment is still paramount to offer a promising but yet unvalidated treatment and that decision is still made on an individual case-by-case basis. It is only that unvalidated treatments cannot be prescribed in the same manner as validated care [16]. The feasibility problem is often linked to the notion of equipoise, conceived as a pre-condition to recruit a patient in a trial [58]: the clinician or patient must have no hint or preference regarding what could be best. But this is putting hints and preferences above scientific evidence. Such a pre-condition could make sense in an explanatory trial, conceived as a device primarily designed to gain knowledge. Equipoise is then understood as a principle which protects the medical interest of voluntary participants by minimally affecting the care decision. Trial inclusion is then limited to patients for whom the individual physician sees no reason to favor one treatment over another. In the case of care trials, the pre-condition of equipoise is mistaken because it is the very medical decisions and care under uncertainty (especially the unjustified use of risky unvalidated interventions) themselves that need to be regulated by the trial to protect the medical interests of the patient. The 2 treatments being offered (and eventually compared) are not “equally” known: the burden of the proof is on the unvalidated treatment. The current notion of equipoise misses the normative role the trial plays in protecting all patients from preventable harm related to the use of unvalidated interventions. According to the framework we propose, while clinical judgment can be used to prescribe a treatment that has been validated as beneficial, it cannot be used to prescribe an experimental treatment that has never been shown to deliver better patient outcomes just as if it were validated care.

The common but dogmatic idea that clinical judgment could replace scientific methods is simply untrue. First, a good medical practice, in the absence of a distinction between safe, known, validated treatments and promising but unproven, experimental treatments, can no longer be defined. Second, what is needed for proper clinical decisions is a comparison of the *same patients* being offered *different* treatments. Without RCTs, the reasons for clinicians to favor one treatment or the other all come from invalid comparisons, experienced in practice, or reported in observational studies, between different outcomes observed in *different* patients managed using the *same* treatment. This is the fallacy of wrong-axis comparisons that we have explained elsewhere, a fallacy which biases all clinical experiences outside RCTs [32]. Third, such case-by-case decisions under uncertainty are unrepeatable, even at the individual clinician level, as we have previously shown for most care trial dilemmas [59]. Unreliable clinical decisions directly lead to forever-unverifiable care.

## First to guide practice; second to gain knowledge

For clinicians, regulation of practice is of the utmost importance, and the sole justification for clinical research is to improve patient outcomes. Unfortunately, it is too often believed (and this may be the source of the mistaken separation between research and practice), that scientific methods can only improve medical care by delivering convincing data at the end of a study. But care in the presence of such uncertainty necessitates a change of practice immediately.

What can we accomplish if there is little hope (at least for now) of reaching a definite answer as to which practice is best? In reality, trial methods such as randomized allocation play an important role in working for the best interest of the patient long before trial results become available. For clinicians, randomized allocation is not primarily a device to balance patient characteristics to make two groups comparable at the end of a trial. For clinicians, randomized allocation is the way to offer a 50% chance of receiving a promising treatment that could be beneficial, but because that treatment could also turn out to be harmful, randomization also offers a 50% chance of avoiding that same unproven treatment. Thus unvalidated care is not prescribed with authority as if it were validated, but only “semi- or 50% prescribed.”

For real-world examples that we have personally experienced, severe acute stroke patients that were recruited in EASI were offered a 50% chance of thrombectomy, and those that allocated thrombectomy benefitted from receiving the treatment long before evidence from randomized trials became available. The participation in EASI also assured that the institution, personnel, and clinicians were ready to offer this treatment 24/7 to the community as soon as the uncertainty was lifted [18]. On the other hand, by participating in FIAT (Flow diversion in the treatment of Aneurysms), 50% of aneurysm patients eligible for flow diversion (a revolutionary approach that can achieve spectacular cures), were protected from previously unknown severe and unanticipated complications, such as delayed arterial thromboses, aneurysmal ruptures, and unexplained hematomas at a distance from the aneurysm, which eventually became known [60]. The immediate benefits for the patient resulting from the change in practice imposed by restricting the use of unproven surgical methods in randomized trials must not be forgotten: this is how > 50% of women could be spared unnecessary total mastectomies in the National Surgical Adjuvant Breast Project (NSAPB) study long before results became available [61]. Care that has not been validated, and especially the use of innovations, must be restricted to care trials, until they are shown to improve patient outcomes. If this is never achieved, then it is for the best, because restricting prescriptions to validated interventions, and using promising but unvalidated interventions only as a 50% chance, balanced by a 50% chance of receiving validated care, is how unnecessary morbidity can be prevented immediately for the patient, as well as on a large scale for future patients.

Care trials re-introduce scientific methods where they belong in practice: to guide care in the presence of uncertainty. Care and research, science, and ethics can thus be reconciled. On one hand, it is impossible that a good medical practice would be incompatible with doctors being able to verify whether their practice does good or harm. On the other hand, clinical research protocols must not be designed in the same manner as laboratory research [7]. Care research is a science of practice, and it must respect the needs and interests of individual patients if results are to be applied in practice.

Practicing outcome-based care under uncertainty necessitates a change in culture and a revision of widely held notions about clinical research and care [15], but the approach is increasingly used and applicable to all medical or surgical domains. We believe the best way to appreciate the clinical value of the care trial methodology is by direct experience. We hope that more physicians and centers will experience the salutary change of practice that occurs when this approach is integrated into care.

## Conclusion

The experience we report suggests that the care trial methodology can be integrated to practice to address various clinical dilemma in multiple and various contexts, including the introduction of therapeutic innovations, emergencies, and wherever clinical uncertainties persist. Care trial methods can be used to optimize patient outcomes in practice in the presence of pervasive uncertainty under the overarching rule to always work in the patient’s best medical interest.

## Data Availability

Data sharing is not applicable to this article as no datasets were generated or analyzed during the current study.
